# Effective management of acne fulminans: Two case reports highlighting fluorescent light energy therapy

**DOI:** 10.1111/jocd.16478

**Published:** 2024-12-12

**Authors:** Jose Luis López Estebaranz, Matteo Bordignon, Roberta Tardugno, Michael Canova Engelbrecht Nielsen

**Affiliations:** ^1^ Dermomedic Clinic University Hospital Fundación Alcorcón Madrid Spain; ^2^ Poliambulatorio San Gaetano Research Unit, Skin Team Thiene Italy; ^3^ Department of Pharmacy – Drug Sciences University of Bari Aldo Moro Bari Italy; ^4^ Fast‐Q Frederiksberg Denmark

**Keywords:** acne fulminans, biophotonic therapy, fluorescent light energy, lesion, scar

## Abstract

**Background:**

Acne vulgaris stands as a prevailing skin condition among adolescents worldwide.

**Objectives:**

The primary aim in addressing acne is to manage it effectively, preventing the occurrence of permanent scarring.

**Materials and Methods:**

This case report outlines the experiences of two adolescent boys grappling with acne fulminans. Initial treatments encompassed isotretinoin, corticosteroids, and doxycycline, until they were discontinued due to their ineffectiveness and the emergence of adverse side effects such as headaches, nausea, and vomiting. Subsequently, fluorescent light energy (FLE) therapy was introduced to tackle these severe cases.

**Results:**

After 4 months of FLE therapy, the patients exhibited significant improvements in their condition. Six months post treatment initiation, both patients have sustained a symptom free state, marked by the absence of active lesions.

**Conclusion:**

FLE therapy effectively treated the acne fulminans cases reported and could be considered as a valid treatment option.

## 1 INTRODUCTION

Acne vulgaris is the most common adolescent skin condition globally, affecting approx. 85% of 12–25 years old in the United States.^1^ The pathogenesis of acne is driven by many interrelated mechanisms, including the presence of *Cutibacterium acnes*, inflammation, increased sebum production driven by androgens,^2^ and hyperkeratinization of the follicular infundibulum.^3^, ^4^ Mild disease is dominated by comedones with some inflammatory pustules. Whereas severe acne is characterized by comedones, papules, pustules, and large, often painful nodular or pustular lesions.^5^ Acne fulminans (AF) is a severe inflammatory acne that manifests suddenly with painful, hemorrhagic pustules and ulceration. Systemic symptoms like fever, polyarthritis, and laboratory abnormalities may also occur. AF represents less than 1% of all acne cases, and predominantly affects Caucasian males, who frequently have a history of acne vulgaris.^6^

The key goal of acne treatment is to treat and control existing lesions whilst minimizing permanent scarring.^7^ Oral systemic treatment, that is, isotretinoin, is indicated for severe cases of acne when nodular lesions or scarring is present.^7^, ^8^ Positive treatment commonly leads to the clearance and remission of acne lesions.^7^ Nevertheless, acne flare‐up, that is, paradoxical worsening of the lesions, not eligibility to standard dose of isotretinoin treatment due to collateral effects or do not reach a satisfying clearance can occur in some patients.^9^

In rare and severe cases, this flare can present as a form of AF, characterized by painful nodular and ulcerative lesions,^10^ which mainly present in adolescent boys. AF is estimated to affect approximately 1% of all acne cases. It is predominantly observed in Caucasian male adolescents (age range: 13–22 years), about 2 years following the earlier onset of acne vulgaris.^11^ It is well‐accepted that acne has a serious impact on a patient's quality of life.^12^ Experiencing worsening acne and emotional distress, highlighting the need for effective methods to address and reassure them.

In light of the above, FLE, an innovative form of photobiomodulation (PBM) can be a promising tool in clinical dermatology as non invasive, in‐office, light‐based method with no systemic side effects for the management of acne cases. The FLE therapy consists of a multiple blue light emitting diode (LED) device coupled with a patented photoconverter chromophore gel.^13^, ^14^, ^15^, ^16^, ^17^ The chromophore when illuminated is capable of photoconverting the blue LED light into all visible spectra wavelengths resulting in a synergistic multiple beneficial effects since each wavelength can penetrate the skin and reach different depths and cellular targets.^13^, ^14^, ^15^, ^16^, ^17^

This case report details two cases of AF in two adolescent boys during treatment with isotretinoin. FLE treatment with known anti‐inflammatory and healing properties efficacious on acne^13^, ^14^, ^15^, ^16^, ^17^ was applied to resolve these difficult cases.

## 2 CASE REPORTS

### 2.1 Case A

**FIGURE 1:**
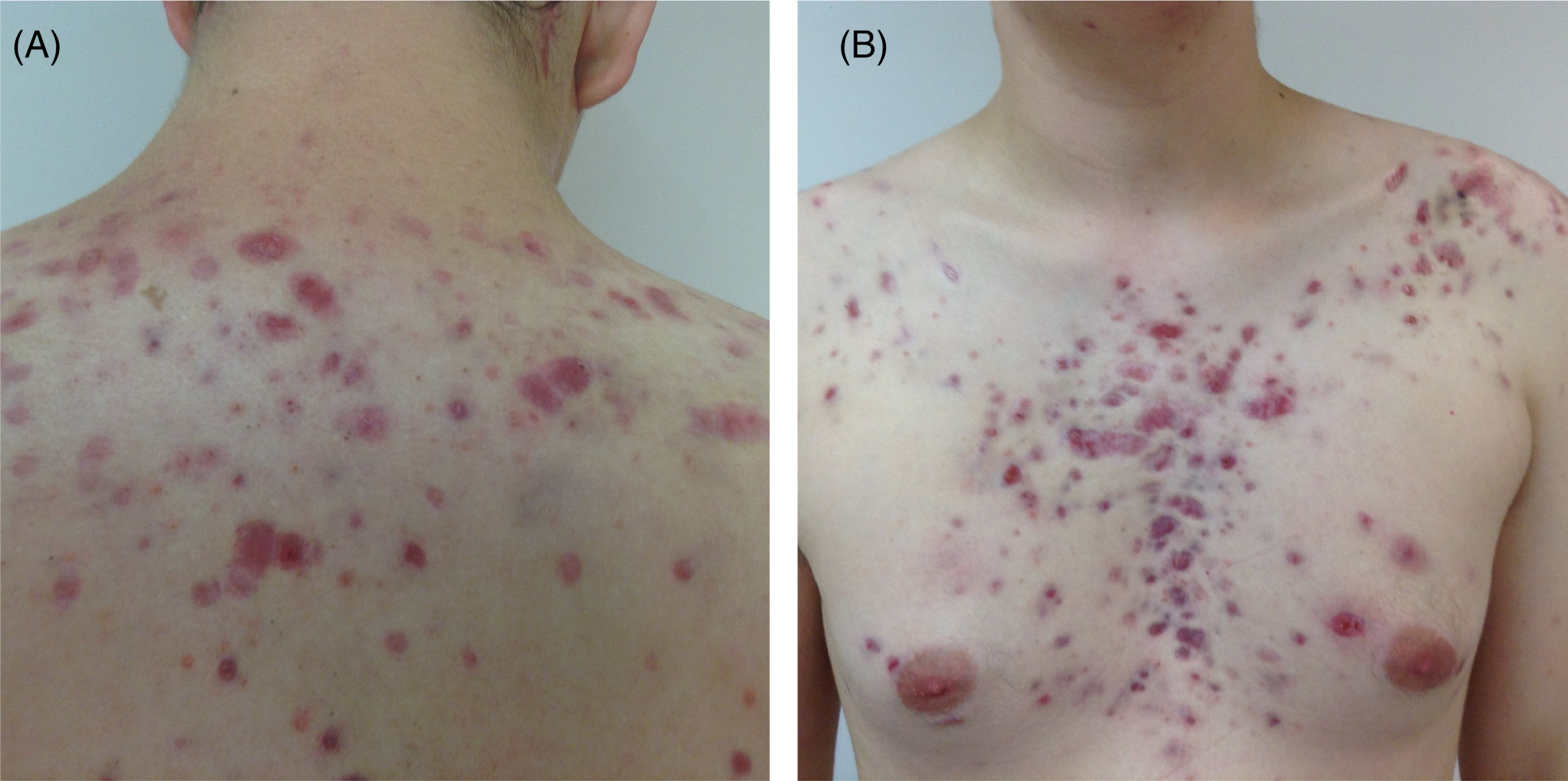
Case A: Acne fulminans initial presentation. (A) Photos taken during the patient's first visit, displaying the chest. (B) Photos taken during the patient's first visit, focusing on the back.

A 17‐year‐old male (75 kg) with Pillsbury grade IV suffering from inflammatory lesions on the face, neck, and trunk for several weeks. The lesions affected most of his face and extensive areas on his chest and back. No systemic symptoms were associated, and no previous medications were taken. On the examination, inflammatory papules and pustules and painful hemorrhagic nodules with hemorrhagic crusts were observed (Figure 1). Open and closed comedones were scarce and inconspicuous. Mild leukocytosis and an elevated erythrocyte sedimentation rate^13^ were observed.

The patient started on a low dose of isotretinoin (Isdiben, Isdin, Barcelona, Spain) 20 mg daily plus oral corticosteroids (Presnisone, Mylan, USA) at a dose of 20 mg. The treatment was discontinued after 3 weeks as the patient experienced strong headache, nausea, and vomiting for periods of several days. A new course using doxycycline (Doxicicline, Normon, Madrid, Spain) was subsequently employed; however also associated with nausea and headache, and a cessation of treatment was selected.

Following the pharmaceutical approach, a biophotonic FLE therapy (Kleresca, FB Dermatology, San Benedetto del Tronto, Italy) was engaged. The therapy consisted of 12 sessions during a period of 6 weeks, at each time point the area of treatment was covered with a chromophore containing gel before activation according to the manufacture's instructions. One month prior to the last treatment, six follow‐up treatments were conducted following the same biweekly treatment schedule as the initial treatment course. The follow‐up treatments proved a substantial improvement of the inflammatory lesions. The red nodules and papulopustular lesions decreased both in number and size. Four months posttreatment initialization, an important improvement was achieved, and no flare‐up of new lesions was experienced. In adjunction to the FLE therapy, a combination of clindamycin 1% and BPO (benzoyl peroxide) 3% (Duac 0.1 by GSK, Madrid, Spain) was applied on the lesions at night and a soft cleanser containing BPO 2.5% (Cetaphil by Galderma, Madrid, Spain) was used for skin cleaning from the beginning of the FLE treatments.

Blood tests (hematological and biochemical parameters) were measured and found to be within the normal ranges.

**FIGURE 2:**
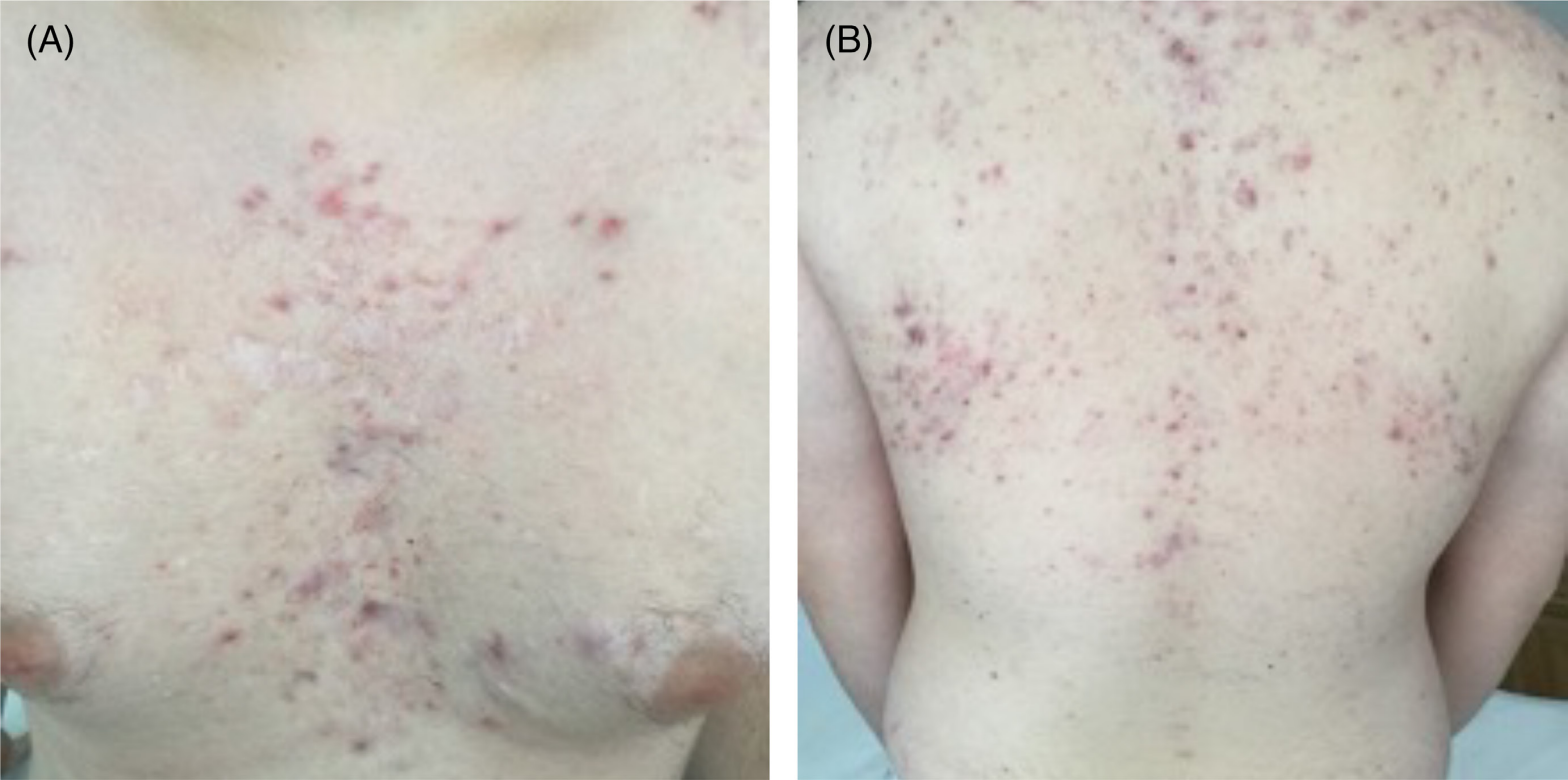
Case A: Eighteen months follow‐up. (A) Follow‐up images captured 18 months later, showcasing the chest. (B) Follow‐up images captured 18 months later, highlighting the back.

At follow‐up at six and 18 months, the patient remained asymptomatic with no active lesions, while some atrophic scars remained visible (Figure 2).

### 2.2 Case B

**FIGURE 3:**
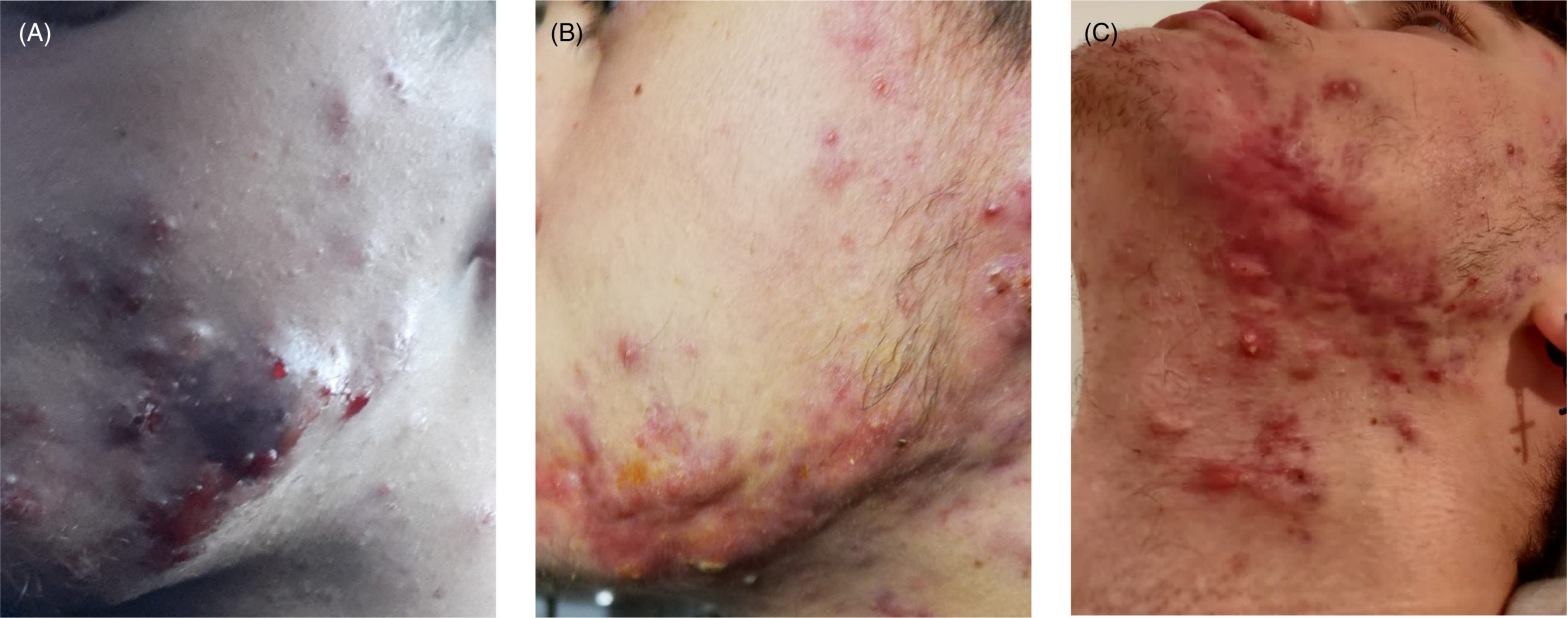
Case B: Acne fulminans treatment progression. (A) Onset of the initial isotretinoin treatment. (B) Cessation of the isotretinoin treatment, followed by the commencement of a combined therapy involving antibiotics and steroids. (C) Images displaying the relapse of acne fulminans.

A 16‐year‐old male (70 kg) with Pillsbury grade III suffering from severe nodulocystic facial acne. The patient started on isotretinoin (Aisoskin, Fidia Farmaceutici Spa, Abano Terme, Italy) 20 mg daily (Figure 3). One month post onset of treatment, the patient developed AF with worsening of the cystic acne with voluminous nodular lesion (Figure 3). The isotretinoin treatment was discontinued, and combination of antibiotics (Zitromax, Pfizer Italia spa, Latina, Italy) and steroids (Deltacortene, Bruno Farmaceutici SPA, Roma, Italy) to prescribed to resolve the AF flareup. One month after the discontinuation of the first isotretinoin treatment the patient engaged in a new treatment regime of isotretinoin with a daily dose of 10 mg. When the dose was increased to 20 mg the patient had a relapse of AF (Figure 3). The isotretinoin was subsequently terminated, and the patient was reemitted to the same combination antibiotic and steroids as previously administrated to resolve the AF flareup.

**FIGURE 4:**
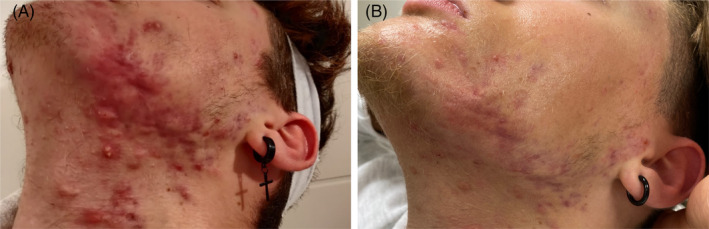
Case B: Acne Fulminans Improvement Post FLE Treatment. (A) Onset of FLE treatment, following the discontinuation of the previous pharmaceutical approach. (B) Four months after the finalization of the FLE treatment, depicting significant improvements.

Succeeding the pharmaceutical approach, FLE therapy was employed. The therapy followed the same standard protocol as for patient case A. No follow‐up treatments were applied to patient case B. Four months post treatment completion, the patient was still in complete remission (Figure 4).

## 3 DISCUSSION

AF is a severe disorder that needs prompt diagnosis and a therapeutic strategy.

Unfotunately, neither standardized guidelines nor a systematic revision of the scientific literature are available regarding AF. Nowadays, the primary treatment options are systemic corticosteroids and oral retinoids.^11^, ^18^, ^19^

The early administration of systemic corticosteroids is recommended due to the anti‐inflammatory and immunosuppressive activities to obtain rapid control of the systemic symptoms, including fever and/or musculoskeletal manifestations. Oral isotretinoin is indicated in AF for its significant ability to reduce abnormal keratinization and sebaceous gland differentiation.^11^, ^18^, ^19^

In selected cases, other drugs can be considered second‐line treatments. Topical agents including corticosteroids, antibacterials, and antiseptics and physical treatments such as debridement, laser, and phototherapies can be successful adjuncts to be taken into consideration.^11^, ^18^, ^19^

The FLE biophotonic platform offers an innovative treatment option for acne, including difficult‐to‐treat moderate to severe cases.^15^, ^16^ Through Stokes shift the light generated by the FLE treatment covers most of wavelengths present in the visible spectrum, combining the nonfluorescent blue light from the LED lamp with the fluorescent green, yellow, orange, and red (415–610 nm) light emitted achieving multiple biological effects.^13^, ^14^, ^15^, ^16^, ^17^ The blue light has been suggested to have an antimicrobial activity on *C. acnes*.^15^ Green light targets the epidermis and upper dermis stimulating fibroblasts and endothelial cells.^13^, ^14^, ^15^, ^16^, ^17^ Yellow light is implicated in the modulation of ATP and fibroblast activity.^13^, ^14^, ^15^, ^16^, ^17^ Orange and red lights with the deepest skin penetration are noted for vascular activation.^13^, ^14^, ^15^, ^16^, ^17^

Edge and colleagues^14^ reported that fibroblasts and keratinocytes responded to the FLE treatment with a reduced output of both TNF‐α and IL‐6 inflammatory cytokines.

FLE anti‐inflammatory effects modulating cytokine responses, possibly justify the beneficial effects experienced on acne inflammatory lesions.^14^, ^20^, ^21^

Furthermore, the stimulation of collagen by fibroblasts and the promotion of angiogenesis mechanisms can play an important role in the skin rejuvenation processes of acne lesions.^14^, ^22^

The FLE treatment has already demonstrated in different clinical studies its very positive safety and tolerability profiles on patients.^13^, ^15^, ^16^ FLE has also been useful in treating peculiar conditions such as acneiform eruption,^23^ *acne conglobata* and *hidradenitis suppurativa* (*acne inversa*).^13^ The cases presented clearly highlight complications in association with systemic treatment of AF with corticosteroids, isotretinoin, and doxycycline. In the presented cases the systemic treatments triggered side effects including nausea, vomiting, hemicrania, and acne flare‐up leading to the discontinuation of the systemic treatments. Clinicians need to understand the potential complications associated with systemic treatment of AF in adolescents and subsequent suspension of those treatments. The FLE therapy showed to be strong non‐invasive treatment option for cases AF presented. In both cases with satisfactory long‐term results considering efficacy and compliance of the patients. FLE could be considered an alternative therapy in some cases of AF that do not tolerate systemic treatment or have recurrence of the lesions.

## AUTHOR CONTRIBUTIONS

Matteo Bordignon and Jose Luis López, Estebaranz: study design, patient selection and clinical assessment. Roberta Tardugno: drafting and revising the original manuscript. Michael Canova Engelbrecht Nielsen: study design and drafting of the original manuscript. All the authors listed have reviewed and approved the final version of the manuscript.

## CONFLICT OF INTEREST STATEMENT

No conflict of interest.

## Data Availability

Data sharing not applicable to this article as no datasets were generated or analysed during the current study.
